# Recombinant DNA technology and click chemistry: a powerful combination for generating a hybrid elastin-like-statherin hydrogel to control calcium phosphate mineralization

**DOI:** 10.3762/bjnano.8.80

**Published:** 2017-04-04

**Authors:** Mohamed Hamed Misbah, Mercedes Santos, Luis Quintanilla, Christina Günter, Matilde Alonso, Andreas Taubert, José Carlos Rodríguez-Cabello

**Affiliations:** 1 G.I.R. Bioforge, University of Valladolid, CIBER-BBN, Paseo de Belén 19, 47011 Valladolid, Spain; 2 Institute of Earth and Environmental Sciences, University of Potsdam, D-14476 Potsdam, Germany; 3 Institute of Chemistry, University of Potsdam, D-14476 Potsdam, Germany

**Keywords:** calcium phosphate, elastin-like recombinamers, hydroxyapatite, mineralization, SN_A_15

## Abstract

Understanding the mechanisms responsible for generating different phases and morphologies of calcium phosphate by elastin-like recombinamers is supreme for bioengineering of advanced multifunctional materials. The generation of such multifunctional hybrid materials depends on the properties of their counterparts and the way in which they are assembled. The success of this assembly depends on the different approaches used, such as recombinant DNA technology and click chemistry. In the present work, an elastin-like recombinamer bearing lysine amino acids distributed along the recombinamer chain has been cross-linked via Huisgen [2 + 3] cycloaddition. The recombinamer contains the SN_A_15 peptide domains inspired by salivary statherin, a peptide epitope known to specifically bind to and nucleate calcium phosphate. The benefit of using click chemistry is that the hybrid elastin-like-statherin recombinamers cross-link without losing their fibrillar structure. Mineralization of the resulting hybrid elastin-like-statherin recombinamer hydrogels with calcium phosphate is described. Thus, two different hydroxyapatite morphologies (cauliflower- and plate-like) have been formed. Overall, this study shows that crosslinking elastin-like recombinamers leads to interesting matrix materials for the generation of calcium phosphate composites with potential applications as biomaterials.

## Introduction

Combination of the specific properties of two materials is often a key to generating a new material, whose properties are superior to those of its individual components, with improved functional performance that can be used for different applications, such as tissue engineering [1]. This perspective can be applied in one of the hottest current research fields, namely control of the formation of calcium phosphate (CP) nanostructures for the generation of biomimetic hybrid materials. Among these CP structures, dicalcium phosphate dihydrate (DCPD), hydroxyapatite (HA) and β-tricalcium phosphate (β-TCP) have attracted attention because of their potential applications [2–3]. HA is a stable crystalline phase that forms the main inorganic component of bone and teeth [2,4]. HA has a nanorod morphology in natural bone, with the individual rods being roughly aligned parallel to one another throughout the collagen matrix [5–7]. β-TCP is a resorbable and degradable synthetic material that can be replaced by naturally re-grown bone tissue [2–3]. As a result, HA and β-TCP have already been used in (composite) materials for bone regeneration [2,8]. Due to the correlation between the (crystal) structure and properties of CP, it is important to be able to control its nanostructures [9–11]. For example, hollow and mesoporous CP particles can be used for drug delivery due to their high specific surface area and three-dimensional porous structures. In addition, osteoblast proliferation and apoptosis are affected by the size and shape of CP. Despite this interest, control of the formation of CP nanostructures with specific characteristics remains a challenge. Although dry methods and high temperature methods are able to produce highly crystalline CP, they also produce aggregated products of large crystal size and low phase purity [9,11]. Moreover, these processes cannot control the morphology and size of the CP generated. Similarly, although wet-chemistry methods can be used to control the size and morphologies of CP under mild reaction conditions, it is difficult to control the crystallinity and phase purity of the synthesized CP nanostructures with narrow size distribution [9,12]. Furthermore, the generation of delicate nanostructures, for example neuron-like morphology of amorphous calcium phosphate (ACP) phase [9,12–13] remains a challenge due to the fast nucleation, aggregation and subsequent anisotropic growth of the crystal faces.

Biomineralization-inspired “soft chemistry” routes provide simple and often rather cheap protocols for the synthesis of complex CP-based hybrid materials with a high application potential [14]. Although organic additives can be used to control the CP mineralization process in aqueous solution, their soluble nature restricts their use in potential applications. To overcome this problem, organic chains are introduced as insoluble additives, for example in the case of a templating approach or in a cross-linked state [15–17]. Following this approach, the most common templates used are Langmuir monolayers and self-assembled monolayers. Mineralization in gel phases has also attracted interest because these gels are easily prepared and provide a high level of control over mineralization [18–19], including the potential to hierarchically generate structured hybrid materials that may, for example, resemble bone tissue [20]. Although extensive research has been conducted on the mineralization field, it is a challenge to integrate CP nanocrystals, different in morphology and composition, into the hydrogel matrices [21].

In order to obtain a hydrogel system that can control the formation of CP, it is important to combine different approaches with each other to overcome the challenges inherent to generating materials with the desired properties. Recombinant DNA technology [22–23] and click chemistry [24–26] are two such approaches. Thus, recombinant DNA technology offers advantages such as reduction in production costs, a time reduction in large-scale bioproduction, close control of the biomacromolecules product sequence (size and uniformity) and high yields [22–23]. Moreover, it provides the possibility to achieve structural complexity by using various bio-inspired materials with distinct mechanical, chemical or biological properties. Biomaterials that can be readily controlled using this approach include the so-called elastin-like polymers (ELPs) or recombinamers (ELRs), which are excellent example of materials that exhibit self-assembly and self-organization [27–29].

The majority of ELPs or ELRs consist of simple amino-acid consensus epitopes that are also present in natural elastin, such as (VPGXG)* (see Table 1 for details of sample nomenclature), where the guest amino acid X can be any of the natural or synthetic L-amino acids except L-proline. ELRs exhibit an intrinsic inverse temperature transition (*T*_t_) in aqueous solution. Thus, below *T*_t_, the ELR chains are highly hydrated, which gives rise to well-solvated polymers in a random coil conformation, whereas above *T*_t_, the ELR chains self-assemble into β-turns, which are organized into a fibrillar morphology [27,30]. Changes to the guest residue X modulate *T*_t_, with the key influence being the polarity of the amino acid side-chain in X. As such, ELRs can be designed with different polarities. This variability provides access to a multitude of related, but different, amphiphilic multiblock ELRs with pre-programmed self-assembling capabilities. Moreover, if the guest residue X carries amino or carboxyl groups, for example in lysine or glutamic acid, different post-synthesis reactions can be performed with the ELRs.

As far as polymer bioconjugation is concerned, the thermoresponsivity of ELRs could be exploited to tune the bioactivity of biological components [13,31–34]. According to this idea, the use of recombinant DNA technology [23,35] allows the combination of ELRs with mineralizing domains to be obtained. One of these mineralization-enhancing segments is the SN_A_15 (DDDEEKFLRRIGRFG)* peptide from salivary statherin that has negative and positive charges stemmed from the side chains of aspartic (D), glutamic (E), lysine (K) and arginine (R) amino acids [36]. The addition of specific functional domains further broadens the application potential of ELRs. The SN_A_15 domain exhibits a high affinity for CP and is able to nucleate mineral phases from calcium- and phosphate-containing solutions [36–37]. Moreover, its α-helical conformation and distinct charge distribution endows this domain a high affinity for HA [36–37].

The use of click reactions – e.g., cycloadditions – to cross-link hybrid elastin-like-SN_A_15 recombinamers synthesized at the gene level, and prepare chemical hydrogels, is considered to be highly successful, since the groups required for cycloaddition – azides and terminal alkynes – are easily introduced [24–26,38]. The reaction conditions for biomedical and pharmaceutical applications are preferably mild and typically performed in water to ensure that the biological structures do not lose their function. Hydrogels can subsequently be synthesized by covalently crosslinking the monomer chains via these azide and cyclooctyne groups by means of a Huisgen [2 + 3] cycloaddition (“click” reaction).

Most of the work performed on ELRs combined with SN_A_15 is about studying their behavior in vitro studies [31–34]. For example, ELR membranes with SN_A_15 epitopes and/or surface topographies have been conducted on the cell morphology, adhesion, proliferation and differentiation, and their potential for dental and orthopedic implants integration during the mineralization process. Although the mineralization activity of ELRs combined with SN_A_15 in soluble state has been investigated so far [13,31], to our best knowledge, there is only one study about the mineralization of ELRs in a hydrogel state [21]. In this study, Li et al. developed mineralized ELR-hydrogels using a polymer induce liquid precursor (PILP) mineralization process where the poly aspartic acids (poly Asp) mimic the role of non-collagenous proteins (NCPs) in biominerals. Although, poly Asp may facilitate the infiltration of ACP into the ELR hydrogel, the interaction of poly Asp with the ELR was not stated. However, to the best our knowledge, the foundations of the impact of SN_A_15 on the mineralization activity of ELR in the hydrogel state have not been provided yet.

In light of the above, the main aim of this work was to evaluate the potential of ELR-based hydrogels for CP mineralization. To that end, recombinant DNA technology was combined with click chemistry to synthesize well-formed and cross-linked hydrogels. Crosslinking of hybrid elastin-like-SN_A_15 chains bearing lysine as amino acid was achieved by means of a Huisgen [2 + 3] cycloaddition.

Two ELRs were used to produce hydrogels: one is based on a simple ELR chain with no specific moiety and the other one is based on a hybrid elastin-like-SN_A_15 recombinamer. In this regard, we are going to focus on the interplay between ELRs, SN_A_15 and CP at the molecular level, in order to discuss the following outstanding inquiries: (1) The role of ELRs during mineralization: How do they interact with the mineral? (2) The role of the SN_A_15 domain: How does it modulate the mineralization of CP by ELRs?

## Experimental

### 1 Materials

All chemicals were purchased from Aldrich Co. and used as received. ((1*R*,8*S*,9*S*)-Bicyclo[6.1.0]non-4-yn-9-ylmethyl succinimidyl carbonate with a purity of 95% was purchased from SynAffix B.V. (Nijmegen, the Netherlands).

### 2 ELR bioproduction

The ELR IK24 and the hybrid elastin-like-SN_A_15 recombinamer H3AH3, with the composition and amino acid sequence shown in Table 1, were synthesized using recombinant DNA technology as described previously [22,31,39]. Cloning and molecular biology experiments for gene construction were performed using standard methods. ELR production was carried out using cellular systems for genetically engineered protein biosynthesis in *E. coli* and purification was performed with several cycles of temperature-dependent reversible precipitation. After purification, ELRs were characterized using matrix-assisted laser deposition ionization time of flight mass spectrometry (MALDI-TOF-MS), attenuated total reflection infrared spectroscopy (ATR-IR) and nuclear magnetic resonance spectroscopy (^1^H NMR).

### 3 Preparation of ELR hydrogels

ELR hydrogels were made by covalent crosslinking of the ELRs via the azide and cyclooctyne groups present in the ELR chains by means of a Huisgen [2 + 3] cycloaddition [24]. The azide and cyclooctyne groups needed for this reaction were introduced directly into the ε-amino group of the L-lysines distributed along the ELR chains. A fraction of the ELR product was functionalized with azide groups as described previously by Lundquist et al. [38]. In this case, all the amino acid groups distributed along the IK24 (25 amine groups) or H3AH3 (31 amine groups) recombinamer chains were modified with an azide group. The other fraction was functionalized with cyclooctyne groups as described previously by González et al. [25]. In this case, 60% of the amine groups distributed along the IK24 or H3AH3 recombinamer chains were modified with a cyclooctyne group.

#### 3.1 Azide derivatization

Briefly, azide groups were introduced by using a diazo-transfer reaction. The reaction was carried out in ultrapure water employing freshly prepared trifluoromethanesulfonic azide (TA) and CuSO_4_.

**TA preparation:** The TA solution was prepared in situ prior to each reaction. NaN_3_ (100 equiv) was dissolved in 14 mL ultrapure water followed by the addition of 5.26 g NaN_3_/19 mL CH_2_Cl_2_, yielding a two-phase mixture. Trifluoromethanesulfonic anhydride (Tf_2_O) (20 equiv) was then added dropwise with continuous stirring at 4 °C. This solution was kept at 4 °C for an hour, and then at room temperature for another hour. The organic phase was separated and washed with a saturated Na_2_CO_3_ solution. This organic TA solution was used without further purification.

**Amine-Azide interconversion:** The ELR was dissolved in ultrapure (Millipore) water at 85 mg/mL and 4 °C. Aqueous Na_2_CO_3_ (1.5 equiv) and CuSO_4_ (0.01 equiv) solutions were added. Methanol (2 mL) was added to act as a phase-transfer agent to transfer TA from CH_2_Cl_2_ to the aqueous phase. The freshly prepared TA solution was added dropwise to the mixture at 4 °C. The azide exchange reaction was performed overnight under an inert atmosphere at room temperature.

The organic solvents were subsequently removed under reduced pressure and the ELR-azide solution was dialyzed against ultrapure water at 4 °C and lyophilized. The product was characterized using ATR-IR spectroscopy, and MALDI-TOF-MS.

#### 3.2 Cyclooctyne derivatization

The amount of (1*R*,8*S*,9*S*)-bicyclo[6.1.0]non-4-yn-9-ylmethyl succinimidyl carbonate required to modify 60% of the amino groups distributed along the recombinamer chain was dissolved in 1 mL of dimethylformamide (DMF). This solution was then added to a solution of the ELR at 25 mg/mL in DMF. The reaction was performed under an inert atmosphere and with continuous stirring at room temperature for 48 hours. The ELR-cyclooctyne product was precipitated by addition of diethyl ether. The white precipitate was washed three times with acetone and dried under reduced pressure. Finally, the ELR-cyclooctyne was dissolved and dialyzed against ultrapure water at 4 °C, lyophilized, and characterized using ^1^H NMR spectroscopy.

### 4 ELR-gel formation

ELR-cyclooctyne and ELR-azide samples were dissolved separately in ultrapure water at 4 °C and 50 mg/mL each. Then, 500 µL of each solution was mixed in a cylindrical mold for one hour at 4 °C (since the chains are more soluble at this temperature, and therefore the two solutions will be well-mixed at the molecular level) and a further hour at room temperature, at which the ELRs are covalently cross-linked to generate the ELR gels. These gels were lyophilized for scanning electron microscopy (SEM).

### 5 Calcium phosphate mineralization

Mineralization was performed by alternative incubation of ELR hydrogels in calcium and phosphate solutions at 37 °C and pH 7.4 for 14 days. Each hydrogel was incubated in 15 mL of a 500 mM aqueous CaCl_2_ solution for two days, followed by rinsing in ultrapure water. The hydrogels were subsequently incubated in 15 mL of a 300 mM aqueous K_2_HPO_4_ solution for another two days then once again rinsed with ultrapure water. This sequence was repeated a maximum of seven times (14 days). Salt concentrations are comparable to those used in other studies [40–44]. To evaluate intermediate stages, samples were isolated at different incubation times (4, 8, and 14 days) by removing and rinsing with ultrapure water followed by drying under vacuum at 37 °C.

### 6 Analysis

#### 6.1 Matrix-assisted laser desorption-ionization time-of-flight mass spectrometry (MALDI-TOF-MS)

The molecular weight (*M*_w_) of the ELRs was determined by MALDI-TOF-MS. Samples were dissolved in ultrapure water at 4 °C. The MALDI-TOF matrix was 2,5-dihydroxyacetophenone (2,5-DHAP). Samples were prepared by dissolving 7.6 mg of 2,5-DHAP in 375 μL of ethanol and mixing with 125 μL of an 18 mg/mL aqueous solution of C_6_H_8_O_7_·2NH_3_. 1 μL of this matrix solution was dispensed onto the MALDI plate along with 1 μL of aqueous ELR solution. The plate was dried in air and mass spectra were recorded using a Bruker autoflex speed instrument with a nitrogen laser (337 nm) operating in positive ion mode with delayed extraction.

#### 6.2 Nuclear magnetic resonance (NMR) spectroscopy

^1^H NMR spectroscopy was performed using an Agilent 400 MHz spectrometer (Agilent technologies) equipped with an Agilent MR console 400 and one NMR probe. For NMR experiments, 15–20 mg of the ELR sample was dissolved in 600 µL deuterated dimethyl sulfoxide (DMSO-*d*_6_). Chemical shifts (δ) are given in ppm. The DMSO signal at δ = 2.54 ppm was used as internal reference [45].

#### 6.3 X-ray diffraction (XRD)

Dry samples were ground in an agate mortar prior to XRD analysis. Powder XRD patterns were recorded for 2 hours using a Bruker D8 Discover A 25 equipped with a Cu K_α_ radiation source (λ = 1.5406 Å), a position-sensitive detector (2θ = 5–70°), and a silicon sample holder. Step size was 0.02°. Phase identification was performed using PCPDFWIN (version 2.2 June 2001 JCPDS).

#### 6.4 Attenuated total reflection infrared spectroscopy (ATR-IR spectroscopy)

Dry samples were ground in an agate mortar and placed directly on the ATR crystal for measurement. IR spectra were recorded using a Bruker Tensor 27 USA spectrophotometer with a diamond crystal. For each spectrum, a 128-scan spectrum was collected from 4000 to 500 cm^−1^ with a 2 cm^−1^ resolution. Spectral analysis was performed using OPUS v4.2 (Mattson Instruments, Inc.).

#### 6.5 Thermogravimetric analysis (TGA)

Dry samples were ground in an agate mortar prior to TGA analysis. TGA experiments were performed using a Mettler-Toledo thermo-balance S.A.E instrument with a horizontal furnace and an automatic gas flow controller. Samples were weighed in a 70 µL aluminum oxide crucible and heated at 20 °C/min under N_2_ purge (40 mL/min) from 50 to 1000 °C followed by an isothermal step at 1000 °C for 10 min.

#### 6.6 Scanning electron microscopy (SEM)

SEM images of the dry ELR/calcium phosphate composites were obtained using an FEI Quanta Field Emission SEM. Samples were fractured mechanically using tweezers prior to mounting on the SEM stub. Images were recorded in low-vacuum mode (7–15 keV, working distance 7.9–13.2 mm), with water as the auxiliary gas. The Ca/P ratios were obtained by energy dispersive X-ray spectroscopy (EDAX Genesis with an Apollo SDD detector, 10 mm). The energy dispersive X-ray (EDX) spectrometer was calibrated for quantitative analysis using Cu and Al for energy and Mo for energy resolution.

## Results

All steps in the production process must be closely controlled to obtain the final hydrogel product with the highest possible yield and correct sequence, and the clickable ELR-based hydrogels studied herein are no exception. This process starts with the gene design, using recombinant DNA technology, and finishes with the final purification steps. The recombinamers are then chemically modified to enable their cross-linking to form a chemical hydrogel.

### Elastin-like recombinamer

Two recombinamers were synthesized using recombinant DNA technology and characterized as previously described [31,39]. The amino-acid sequence of the corresponding building blocks (the elastin-like recombinamer IK24 and the hybrid elastin-like-SN_A_15 recombinamer H3AH3) are shown in Table 1. IK24 is essentially a 24-fold repetition of the unit (VPGIG)_2_(VPGKG)(VPGIG)_2_ in which the hydrophilic blocks (VPGKG) are distributed among the hydrophobic blocks (VPGIG) [39]. This ELR displays a *T*_t_ at about 31.5 °C. The second recombinamer, H3AH3, shows a more complex sequence with *T*_t_ of about 27 °C [31]. It has a triblock architecture with two end blocks made of a modified version of IK24 and a central (VPAVG)_20_ block. In addition to the IK24 sequence, the two end blocks bear six SN_A_15 epitopes, which are regularly interdispersed along the block chain (Table 1). The main advantage of recombinant DNA technology is the ability to control the distribution of lysines and the SN_A_15 bioactive domain along the recombinamer chain.

**Table 1:** ELRs used in the current study.

ELR	Amino acid sequence^a^	*M*_w_ (Da)

IK24	MESLLP[[(VPGIG)_2_(VPGKG)(VPGIG)_2_]_24_V	51996.5 ± 11.3
H3AH3	MESLLP[[(VPGIG)_2_(VPGKG)(VPGIG)_2_]_2_DDDEEKFLRRIGRFG[(VPGIG)_2_(VPGKG)(VPGIG)_2_]_2_]_3_(VPAVG)_20_[[(VPGIG)_2_(VPGKG)(VPGIG)_2_]_2_DDDEEKFLRRIGRFG[(VPGIG)_2_(VPGKG)(VPGIG)_2_]_2_]_3_V	71430.7 ± 12.9

^a^Where, D = L-aspartic acid, E = L-glutamic acid, K = L-lysine, F = L-phenylalanine, L = L-leucine, R = L-arginine, I = L-isoleucine, G = glycine, V = L-valine, P = L-proline, M = L-methionine and S= L-serine.

### Elastin-like recombinamer hydrogel formation

**Figure 1:**
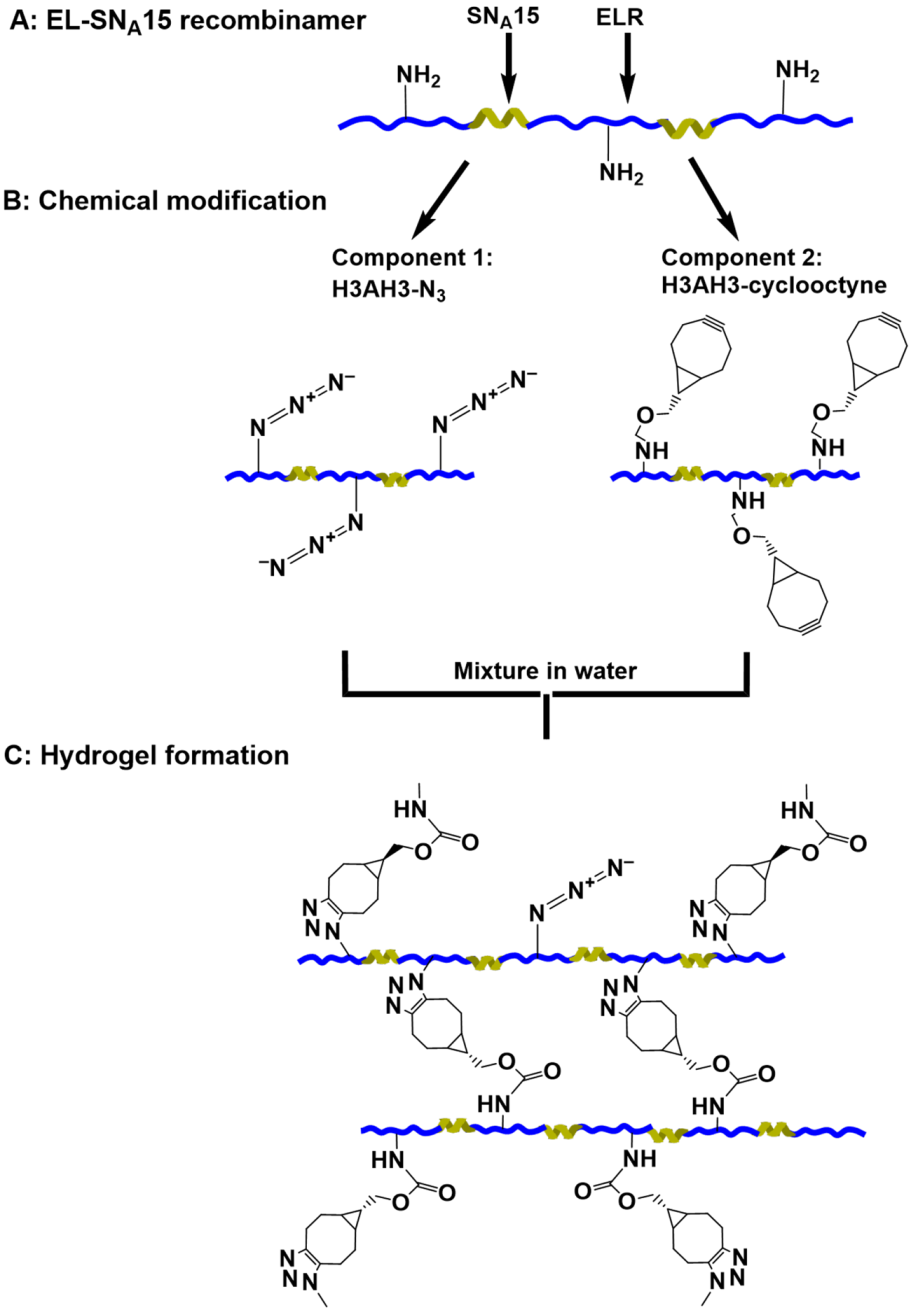
Schematic representation of (A) the H3AH3 recombinamer, (B) azide- (component 1) and cyclooctyne-derivatized H3AH3 (component 2), and (C) formation of the hydrogels via Huisgen [2 + 3] cycloaddition (“click” reaction).

Click reactions are a class of reaction that can often also be performed in biological fluids. These reactions are based on pairs of functional groups, for example azide and alkyne, which rapidly and selectively react with one another under mild conditions [24–25,38]. Functional groups such as the ε-amine groups present in the lysine side chains of IK24 and H3AH3 ELRs can be modified to bear two complementary chemical functions. This process is shown in Figure 1 for H3AH3 as an example. Half of the ELR product was functionalized with azide groups by treatment of the amine residues with triflyl azide (TfN_3_), as described elsewhere [38] (component 1, Figure 1B). The other half was modified by adding cyclooctyne groups by amidation, with the ε-amino groups of the lysine residues being converted into cyclooctyne bearing an activated carboxyl group (*N*-succinimidyl ester), as described elsewhere [25], to form component 2 (Figure 1B).

Azide derivatization of ELRs is indicated by an increase in their respective *M*_w_ in the MALDI-TOF-MS spectra (Supporting Information File 1, Figures S1, S3), and a characteristic absorption band at 2100 cm^−1^ in their ATR-IR spectra (Supporting Information File 1, Figures S2, S4) [46]. Chemical transformation of the ε-amine into the respective cyclooctyne derivative was confirmed by ^1^H NMR spectroscopy (Supporting Information File 1, Figures S5, S6). The ^1^H NMR spectra show signals at 2.91, 4.02, and 7.04 ppm, which can be assigned to the methylene group adjacent to the carbamate group, the methylene group adjacent to the cyclopropyl and carbamate groups, and to the proton bonded to nitrogen from the carbamate group, respectively.

The hydrogel was formed by mixing an aqueous solution of the azide-modified ELR (component 1) with an equivolume aqueous solution of the same ELR, but modified with cyclooctyne (component 2). Upon mixing, the ELRs undergo cross-linking via a Huisgen [2 + 3] cycloaddition, thus giving rise to hydrogel formation (Figure 1C).

**Figure 2:**
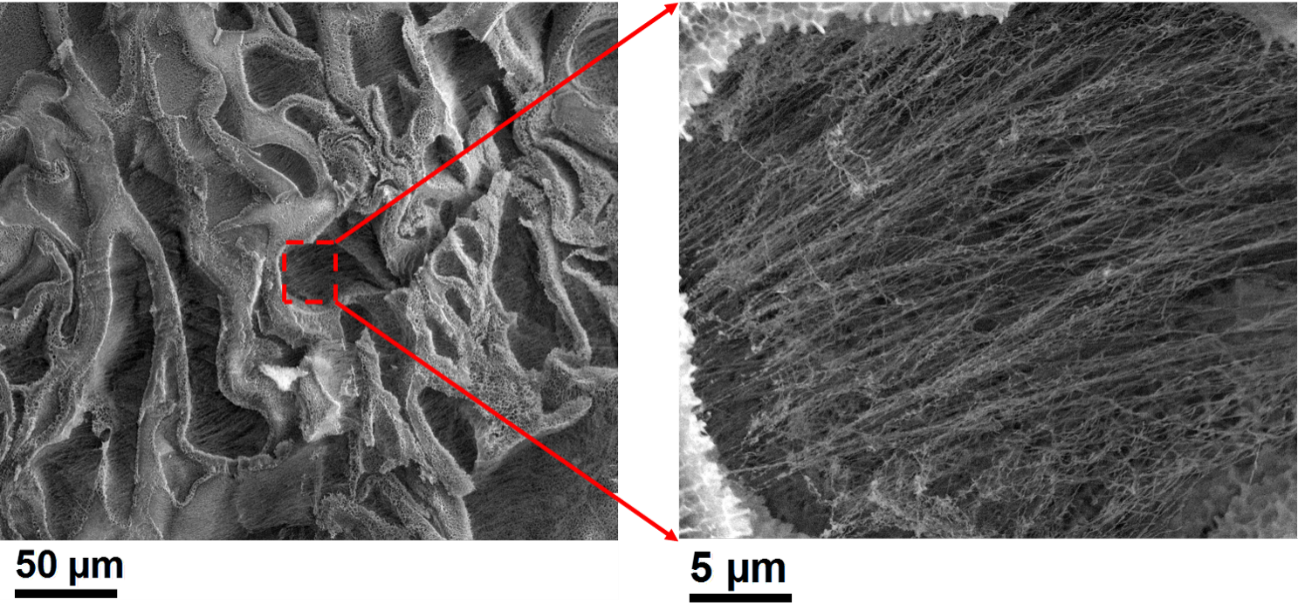
H3AH3 ELR hydrogel morphology observed by SEM. A high magnification image of the red framed area is shown in the micrograph on the right.

SEM images of the cross-linked IK24 and H3AH3 ELR-based hydrogels are shown in Supporting Information File 1, Figure S7 and Figure 2, respectively. The high magnification image reveals the existence of a fibrillar morphology. It is known that ELRs undergo hierarchical self-assembly producing a fibrillar structure [27,30,47]. For example, poly(VPGVG) and its analog poly [*f_v_*(VPGVG), *f_x_*(VPGKG)] (0.1 ≤ *f_x_* ≤0.2, *f_v_* + *f_x_* = 1) interact hydrophobically and self-assemble into nanofilaments that align in parallel, forming fibrillar structure of several hundred nanometres in diameter [47]. Thus, the SEM micrographs (Figure 2 and Supporting Information File 1, Figure S7) clearly show that the chains after cross-linking inside the IK24 and H3AH3 hydrogels are composed of thin, rather homogeneous, fibres that often appear to have some order or, at least, a somewhat parallel organization. This fibrillar structure resembles that of physical elastin-based hydrogels and natural elastin [27,30].

### Calcium phosphate mineralization

**Figure 3:**
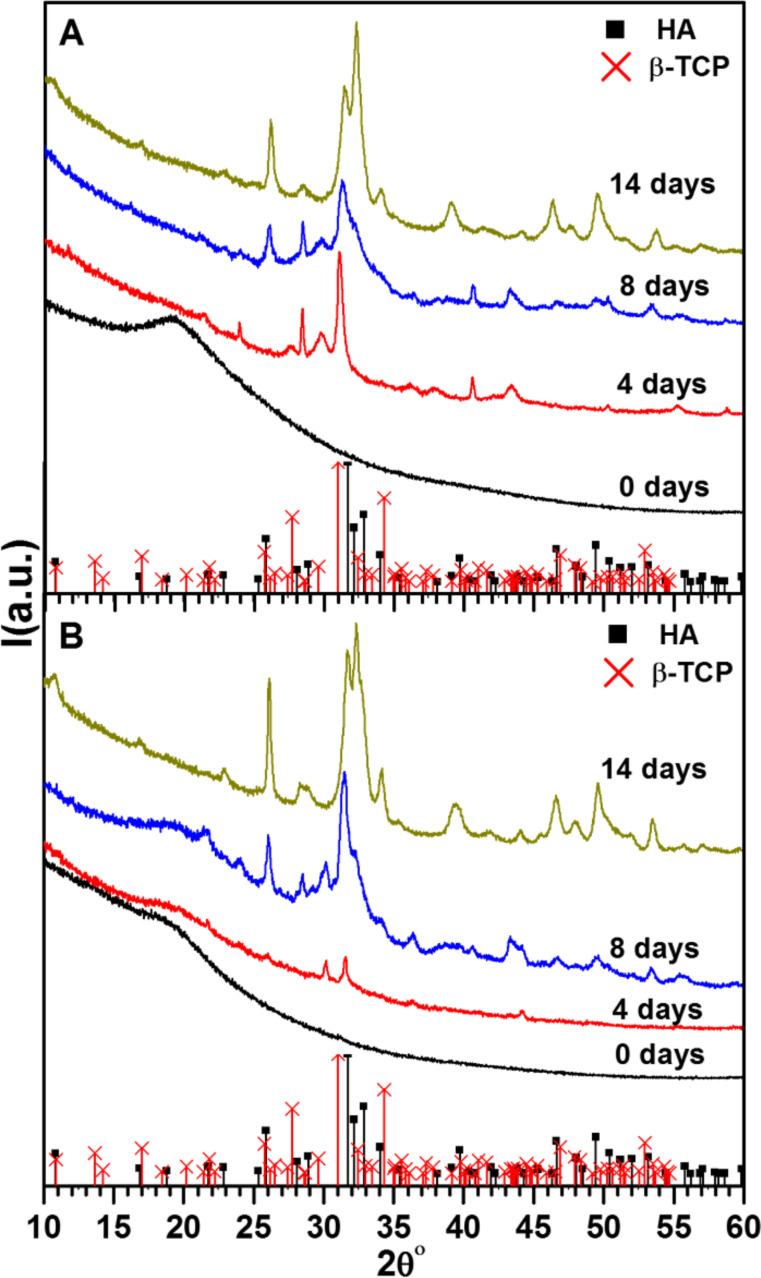
XRD patterns of mineralization products. (A) IK24 hydrogel, and (B) H3AH3 hydrogel after different incubation times. The XRD patterns of HA and β-TCP (bars) have been included for comparison.

The solid-state structure of the precipitates obtained from the mineralization reaction was investigated by XRD. Figure 3A shows the XRD patterns obtained from the IK24 ELR-based hydrogel (no SN_A_15 domain) after different incubation times. Due to the large fraction of organic matter and the generally rather low order of many CPs, all patterns are rather noisy; this is consistent with the literature [14,48]. After 4 days of mineralization (two days in calcium chloride followed by two days in potassium phosphate solution), the diffraction patterns show reflections at 27.47, 28.43, 29.73, 31.08, 36.14, 37.89, 40.61, 43.45, 50.28, 55.28, and 58.81° 2θ. After 8 days of mineralization, and using the same alternating immersion process, new reflections at 26.05, 31.31, 31.74, 32.20, 49.37, and 53.46° 2θ emerge. After 14 days of mineralization, XRD patterns assigned to the typical pattern of HA (JCPDS 01-072-1243) are found.

Figure 3B shows XRD patterns obtained from the hydrogel H3AH3 (with the SN_A_15 domain). After 4 days of mineralization, two peaks at 30.14° and 31.55° 2θ are observed. After 8 days of mineralization, the collected pattern is similar to that obtained for the hydrogel lacking the SN_A_15 domain (IK24). After 14 days, XRD indicates the presence of HA (JCPDS 01-072-1243). The broad and rather non-descript signals that are also observed in many samples are presumably due to the presence of the ELR gel phase, which is still present in these materials.

**Figure 4:**
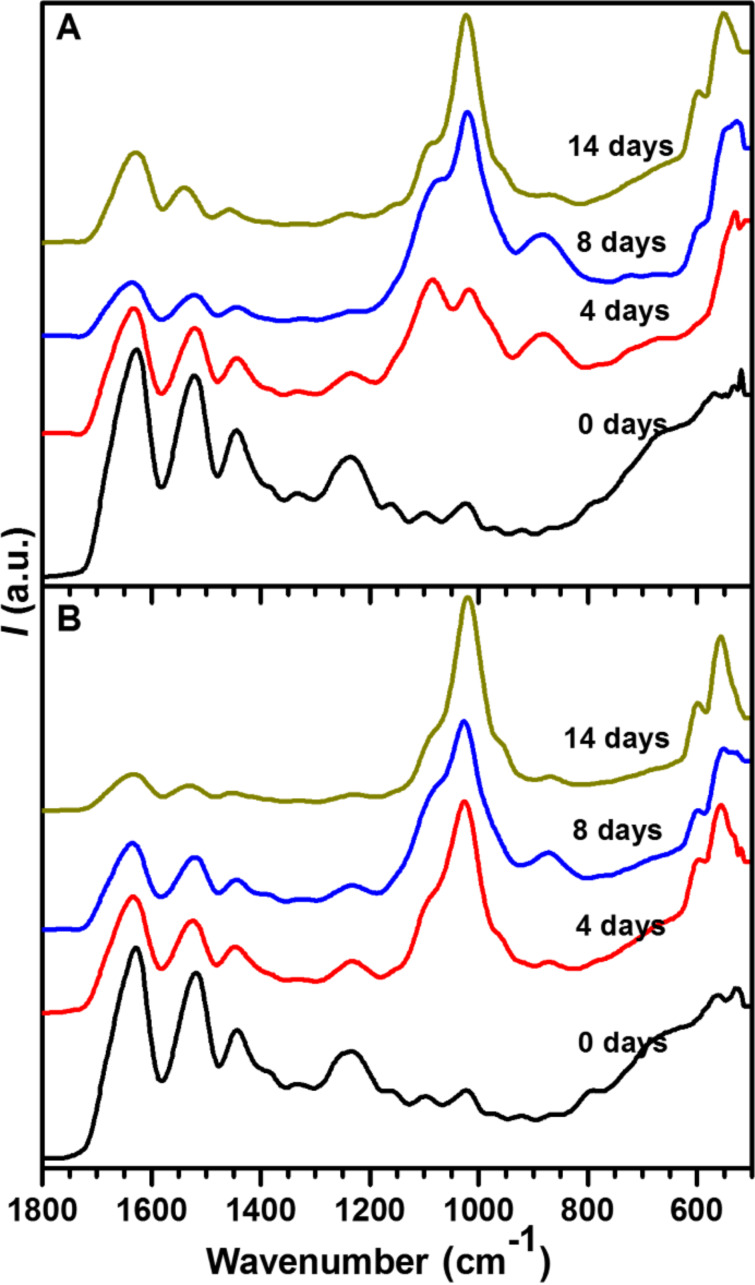
ATR-IR spectra of the mineralized hydrogels: (A) IK24 hydrogel, and (B) H3AH3 hydrogel after different incubation times.

Figure 4 shows the IR spectra of the IK24- and H3AH3-based hydrogels, with the bands at around 1628, 1518, and 1233 cm^−1^ being attributed to amide I (C=O stretching), amide II (mainly C–N stretching), and amide III (N–H in plane deformation) vibrations, respectively [49–50]. In addition, the bands at around 1333 and 1097 cm^−1^ are assigned to δ(CH) and N–C_α_ vibrations, respectively. The signals between 1400 and 1500 cm^−1^ are attributed to CH_3_ asymmetric bending, CH_2_ scissoring, and COO^−^ symmetric stretching vibrations. The band at around 1018 cm^−1^ is assigned to nonstoichiometric apatites containing HPO_4_^2−^ ions, whereas the shoulder at around 1084 cm^−1^ is assigned to the ν_3_(PO_4_)^3−^ vibration in stoichiometric HA [51–53]. The band at around 881 cm^−1^ is assigned to HPO_4_^2−^, most likely due to the presence of DCPD. The bands at around 600 and 550 cm^−1^ are assigned to ν_4_(PO_4_)^3−^ bending mode.

**Figure 5:**
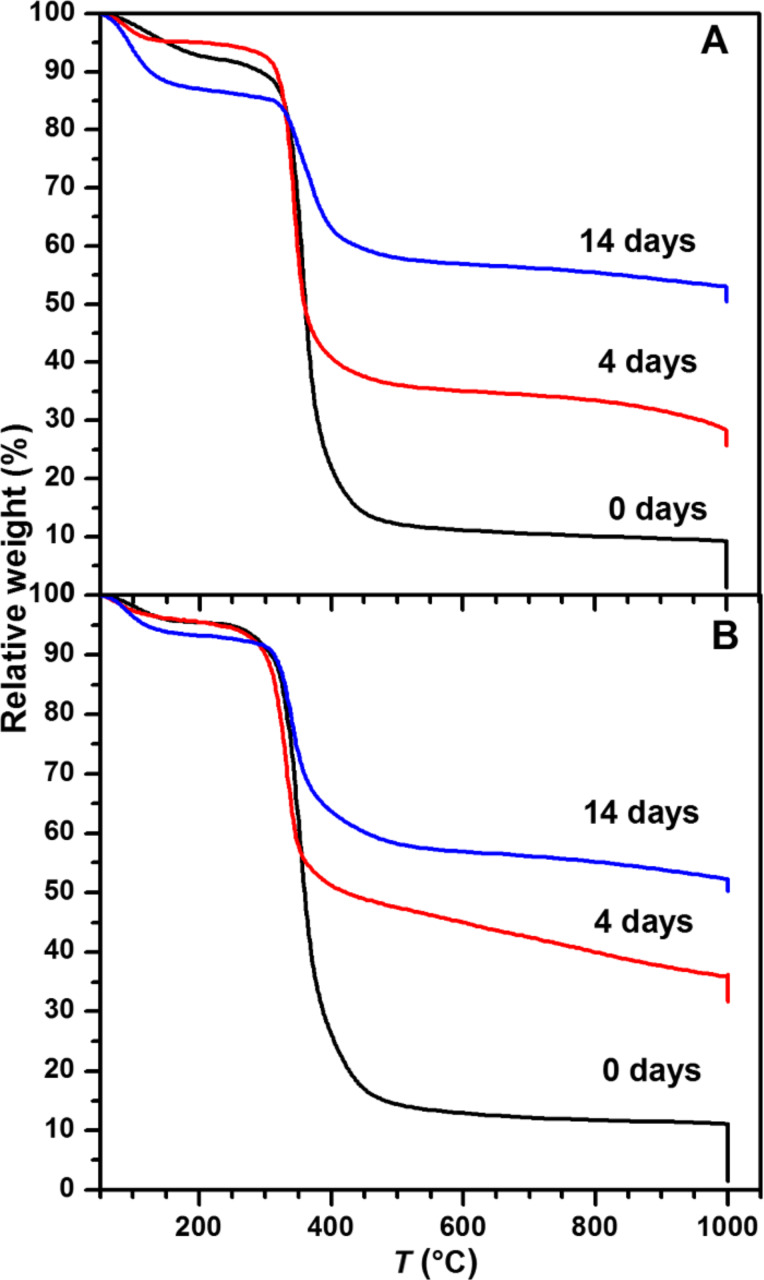
TGA curves for (A) IK24 hydrogels mineralized for 0, 4, and 14 days, respectively, and (B) H3AH3 hydrogels mineralized for 0, 4, and 14 days, respectively.

Figure 5 shows the thermogravimetric analysis (TGA) curves obtained for the ELR hydrogels after mineralization and drying. The first weight loss (around 5–15%), which occurs at between around 80 and 150 °C is assigned to water evaporation. The weight loss in the range 250–450 °C can be attributed to ELR degradation and to further water loss from the CP mineral phase. The relative weight concomitant with this process allows an estimation of the final relative CP content in the hydrogels as a function of mineralization time. At 4 days, the relative weight is about 29% and 36% for IK24 and H3AH3 hydrogels, respectively, whereas after 14 days it is about 53% for both ELR hydrogels. This is in contrast to the pure hydrogels before mineralization, which lose 90% of their initial weight up to 1000 °C. The remnant 10% of the pure hydrogel are all decomposed when they are subjected to an isothermal process at 1000 °C. As such, there is no significant difference between the IK24- and H3AH3-based hydrogels.

**Figure 6:**
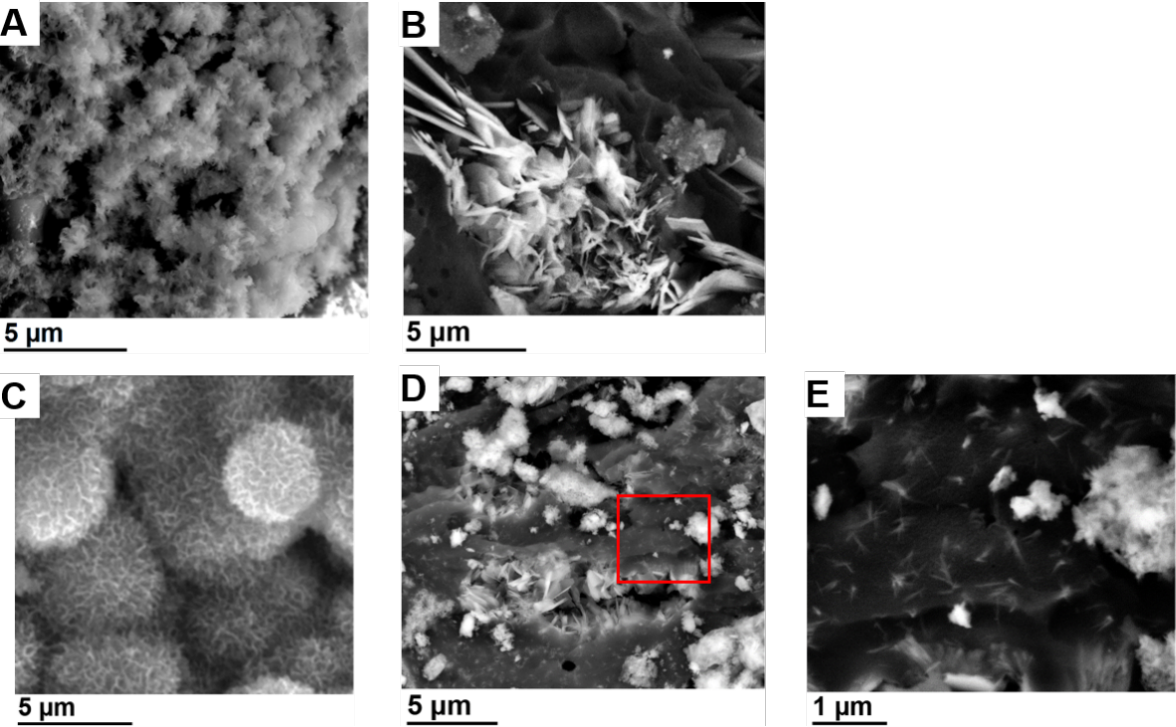
SEM images of the hydrogels after mineralization: (A) IK24 and (B) H3AH3 hydrogels after 4 days of mineralization. (C) IK24 hydrogel after 14 days of mineralization. (D) H3AH3 hydrogels after 14 days of mineralization. (E) High magnification SEM micrograph of the red framed region in D.

Figure 6 shows scanning electron micrographs of CP formed within the hydrogels after different mineralization times. After 4 days of mineralization, IK24-based hydrogels exhibit a spherical structure somewhat resembling a microscale cauliflower morphology (Figure 6A). When the mineralization time increases, the morphology remains the same, but the features increase in size. After 4 days, the individual “cauliflower” is on the order of 1–3 µm, whereas after 14 days their diameter is around 4–8 µm (Figure 6C) and they densely populate the entire material. In the case of the H3AH3 hydrogels, a plate-like morphology is formed after incubation for 4 days (Figure 6B). In addition to the spherical aggregates (Figure 6D and Supporting Information File 1, Figure S8A) generated by the H3AH3-based hydrogels after incubation for 14 days, the calcium phosphate also forms plates (Figure 6D, and Supporting Information File 1, Figure S8B) with lengths of around 1–5 µm. Figure 6E shows, at high magnification, the dispersed plates-like HA that are embedded into the ELR hydrogel matrix and distributed over the entire material, which could result from the interaction of calcium and phosphate ions with SN_A_15. These plates are intertwined and strongly clustered (Supporting Information File 1, Figure S8B). As a result, it is difficult to quantify their sizes in detail. As many of these aggregates appear to grow from a central point, they have a petal-like morphology that is clearly different from the previous spherical cauliflower-type precipitates. As such, different morphologies (plate-like and spherical-like structures) are simultaneously generated in the H3AH3 hydrogels.

Energy-dispersive X-ray spectroscopy (EDXS, Supporting Information File 1, Table S1) shows that all samples contain Ca, P, C, O, and also K and Cl impurities from the initial reagents (CaCl_2_ and K_2_HPO_4_). In the case of IK24, the Ca/P ratio increases from 1.17 to 1.52 as the incubation time increases from 4 to 14 days. In the case of the H3AH3 hydrogel, the Ca/P ratio also increases from 1.12 to 1.78 as the incubation time increases from 4 to 14 days. Overall, the EDXS data show an increase in the Ca/P ratio with increasing reaction time, irrespective of the type of hydrogel used for mineralization.

## Discussion

The combination of two or more materials can give rise to the formation of a multifunctional hybrid system with properties superior to those of each component separately. This combination depends on both the properties of each component and on the way in which they are assembled so that they can work cooperatively. Recombinant DNA technology allows the overall architecture of recombinamers to be controlled, thus making recombinant proteins interesting for a variety of reasons [22]. Among others, their behavior in aqueous media can be adjusted by appropriate design of the amino acid sequence, addition of bioactive domains – e.g., SN_A_15 – and by the solution composition. For example, temperature changes are an efficient means of self-assembling and disassembling ELPs and ELRs [27,29]. Recently, the self-assembly properties of ELRs have been combined with the mineralization capacity of SN_A_15 using such a biotechnology approach [13]. In that study, Misbah et al. demonstrated that ELRs cannot control the formation of CP in the absence of SN_A_15. In addition, the self-assembly properties of ELRs have a marked influence on the mineralization activity of SN_A_15. These complementary functionalities give rise to the formation of fibre- or petal-like HA, in addition to amorphous CP with a neuron-like morphology.

In the ELRs used in this current work (IK24 and H3AH3), the lysine amino acids are distributed in a regular manner along the recombinamer chains. In addition, the nucleating SN_A_15 domains in H3AH3 are also distributed along the recombinamer chain, thus giving rise to the formation of hybrid elastin-like statherin recombinamers. The IK24 and H3AH3 chains are cross-linked via the Huisgen [2 + 3] cycloaddition to form a hydrogel matrix containing well-defined calcium phosphate binding domains. The success of this cycloaddition reaction with ELRs is mainly dependent on the success of the recombinant DNA technology used to distribute the lysine amino acid residues along the ELR chain in a regular manner. These lysine amino acid residues are easily modified with cyclooctyne and azide groups (Figure 1, Supporting Information File 1, Figures S1–S6). Moreover, the cross-linking reaction is easily performed in water, which is an important condition for biomedical applications as it avoids problems with toxic reagents.

XRD provides two significant results, indicating that the ELR-based hydrogels affect the CP mineralization process. The first one is the phase-incubation time dependence. After mineralization for 4 days, the peaks observed at 29.73, 31.08, 36.14, 37.89, and 50.28° 2θ can be assigned to β-TCP phase (JCPDS 01-070-2065). β-TCP is normally synthesized at high temperature, although there are a few examples of β-TCP formation under milder conditions. For example, β-TCP can be synthesized at room temperature in methanol from CaHPO_4_ and ACP precursor phases [54], or in ethylene glycol [55]. Two studies using aqueous solutions also reported the formation of β-TCP. CP nanoparticles prepared in the presence of Aliquat 336 also seem to consist of β-TCP, at least partly [56]. Poly(acrylic acid) hydrogels immersed in a phosphate solution were also calcified by β-TCP [57]. However, the other intense XRD peaks assigned to β-TCP at 32.42 and 34.33° 2θ are not observed. Moreover, the IR band at around 881 cm^−1^ assigned to HPO_4_^2−^ [41,58] suggests the presence of DCPD. Since the Ca/P ratio calculated by EDX is around 1.1 for both hydrogels after mineralization for 4 days, DCPD formation cannot be ruled out [14,48]. In order to clarify this point, other mineralization conditions and characterization techniques are being studied. With increasing the incubation time to 8 days, new reflections at 26.05, 31.31, 31.74, 32.20, 49.37, and 53.46° 2θ indicate the HA formation (JCPDS 01-072-1243). Therefore, the mineralization is kinetically driven where a transient phase β-TCP is formed and eventually transformed into HA. It is worthwhile mentioning that CP precipitation in collagen matrices is also kinetically driven, with transient precursors such as ACP, octacalcium phosphate (OCP), and even TCP [5–7].

The second observation is the SN_A_15-functionality dependence, where the CP phases formed after 4 days of incubation show clear difference between the two hydrogels (see XRD patterns in Figure 3). The CP phase shows stronger reflections in the case of the IK24 hydrogel than in the H3AH3 hydrogel (functionalized with SN_A_15), suggesting that SN_A_15 inhibits the spontaneous transformation of ACP into the crystalline phase. This behavior may be related to the CP morphologies generated by this family of ELRs at soluble state. In a previous study [13], the building block H3 [(IK)_2_-SN_A_15-(IK)_2_] inhibits the transformation of ACP into the crystalline phase in solution, thus giving rise to the formation of a neuron-like morphology. In the confined state (IK24 and H3AH3 hydrogels in the current work) this effect seems to be observed after incubation for 4 days, with formation of the HA phase being supressed and other CP phases being generated. Nevertheless, after incubation for 14 days the CP formed gives rise to the formation of spherical- or petal-like HA. Moreover, not only the phases and morphologies of the CP can be controlled, but also the mineral content. According to Figure 5, after 4 days, mineralized hydrogel matrices with poorly crystalline CP show a mineral content of about 29% and 36% for IK24 and H3AH3 hydrogels, respectively. After 14 days, mineralized hydrogel matrices with HA of different morphologies and high mineral content (about 53%) are achieved for both hydrogels.

The present work shows the merit of using the in situ-inhibition CP approach, where SN_A_15 moieties are regularly distributed along the hydrogel matrix for controlling the CP mineralization process. The combination of the self-assembly properties of ELRs with the mineralization capacities of SN_A_15 gives rise to the formation of plate-like HA, different from the spherical cauliflower morphology formed by IK24. Surprisingly, the ELR H3AH3 at the insoluble state controls the formation of the plate-like of crystalline CP phase (HA), whereas its building block H3 controls the formation of neuron-like morphology of ACP phase at the soluble state [13].

The approach used in this work, which avoids the need for substances that may harm the human body (methanol, ethylene glycol, surfactants) [54–56], shows the merit of controlling the generation of hybrid materials for potential biomedical applications. At the same time, these materials, at the insoluble state, have the ability to control the formation of CP in terms of both phase and morphology, different from the ones formed at the soluble state.

## Conclusion

The present work shows the merit of using the in situ-inhibition CP approach, via the hybrid elastin-like-SN_A_15 recombinamer hydrogel matrix. This merit depends on the combination of ELRs properties with the CP binding capacities of SN_A_15, and on the integration of two synthesis approaches (recombinant DNA and click chemistry) to form advanced biocompatible soft and hybrid materials. Recombinant DNA technology allows the distribution of lysine amino acids and the SN_A_15 domains along the recombinamer chain to be carefully controlled. Cyclooctyne and azide groups can then be introduced successfully by functionalizing these lysine groups. The resulting functional groups are easily cross-linked via click chemistry in an aqueous medium to form a hydrogel matrix. This hydrogel matrix has a marked impact on the mineralization of calcium phosphates. In absence of the SN_A_15 nucleation inhibitor, cauliflower-like HA is formed. In contrast, plate-like HA grows in the presence of SN_A_15. The soluble state of the ELRs shows the merit of generating delicate nanostructures, such as neuron-like morphology [13]. Yet, their soluble nature could restrict their applications for tissue engineering as compared to its hydrogel state. When hydrogel matrices are used, different HA morphologies are obtained after incubation for 14 days (spherical or plate-like) that are not easy to obtain in the soluble state.

In conclusion, the combination between recombinant DNA and click reaction approaches has resulted in the generation of well-programmable and controlled ELR hydrogels to regulate the formation of calcium phosphate nanostructures.

## Supporting Information

File 1 MALDI-TOF spectra, NMR spectra, ATR-IR spectra, SEM micrographs, EDXS analysis are presented.
