# Supplementary material for: Recombinant DNA technology and click chemistry: a powerful combination for generating a hybrid elastin-like-statherin hydrogel to control calcium phosphate mineralization
Source: Beilstein J Nanotechnol. 2017 Apr 4;8:772–83. doi: 10.3762/bjnano.8.80 (PMC5389180; doi:10.3762/bjnano.8.80)
Supplement: File 1 — MALDI-TOF spectra, NMR spectra, ATR-IR spectra, SEM micrographs, EDXS analysis are presented. [file Beilstein_J_Nanotechnol-08-772-s001.pdf]

Recombinant DNA technology and click chemistry: a powerful combination for generating a hybrid elastin-like-statherin hydrogel to control calcium phosphate mineralization

Mohamed Hamed Misbah[1], Mercedes Santos[1], Luis Quintanilla[1], Christina Günter[2], Matilde Alonso[1], Andreas Taubert[3] and José Carlos Rodríguez-Cabello*[1]

Address: [1]G.I.R. Bioforge, University of Valladolid, CIBER-BBN, Paseo de Belén 19, 47011 Valladolid, Spain, [2]Institute of Earth and Environmental Sciences, University of Potsdam, D-14476 Potsdam, Germany and [3]Institute of Chemistry, University of Potsdam, D-14476 Potsdam, Germany

Email: José Carlos Rodríguez-Cabello - roca@bioforge.uva.es

* Corresponding Author

MALDI-TOF spectra, NMR spectra, ATR-IR spectra, SEM micrographs, EDXS analysis are presented

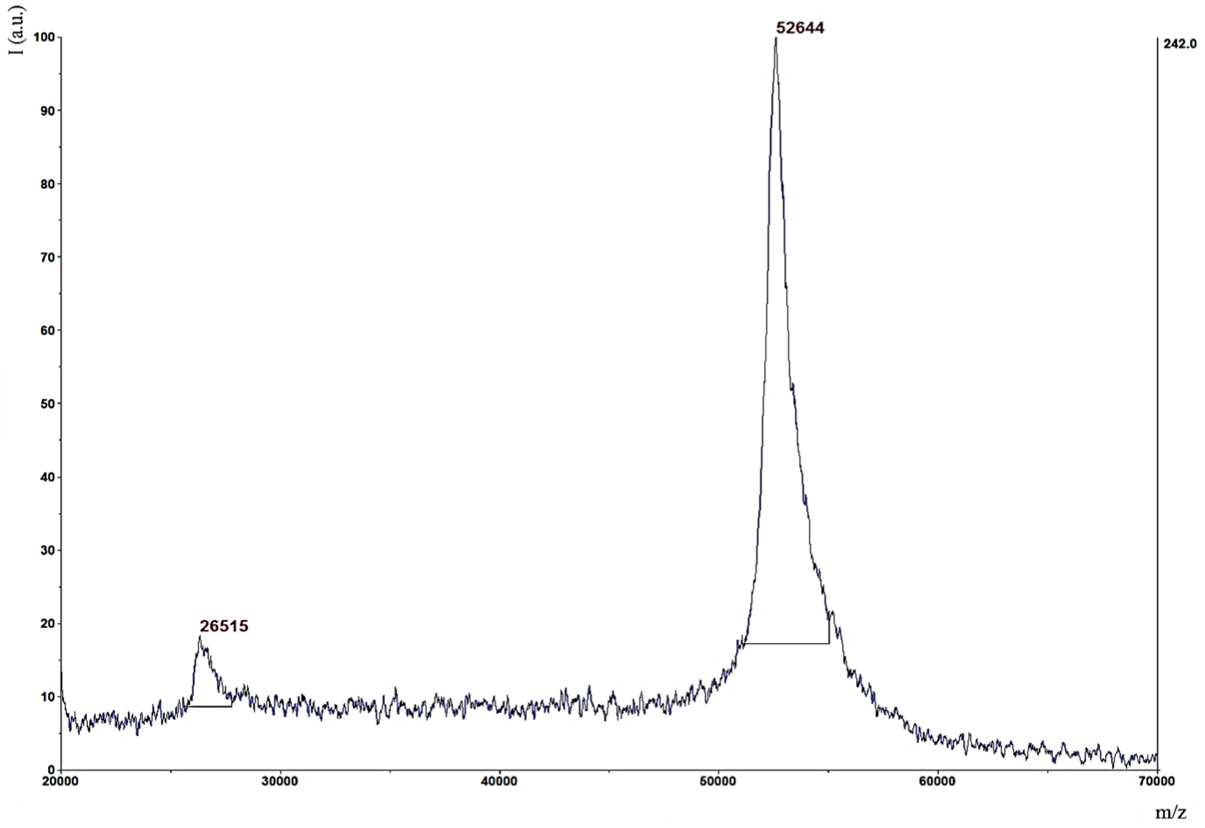

Figure S1: MALDI-TOF of the IK24 ELR modified with azide group.

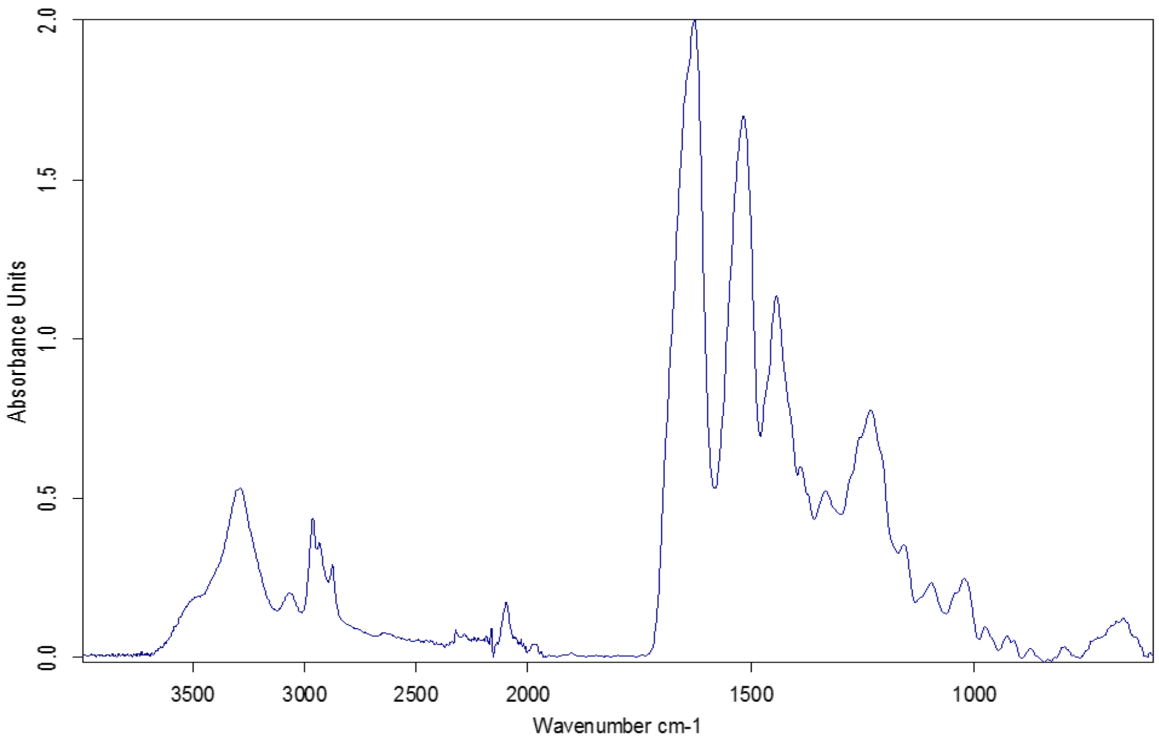

Figure S2: ATR of the IK24 ELR modified with the azide group.

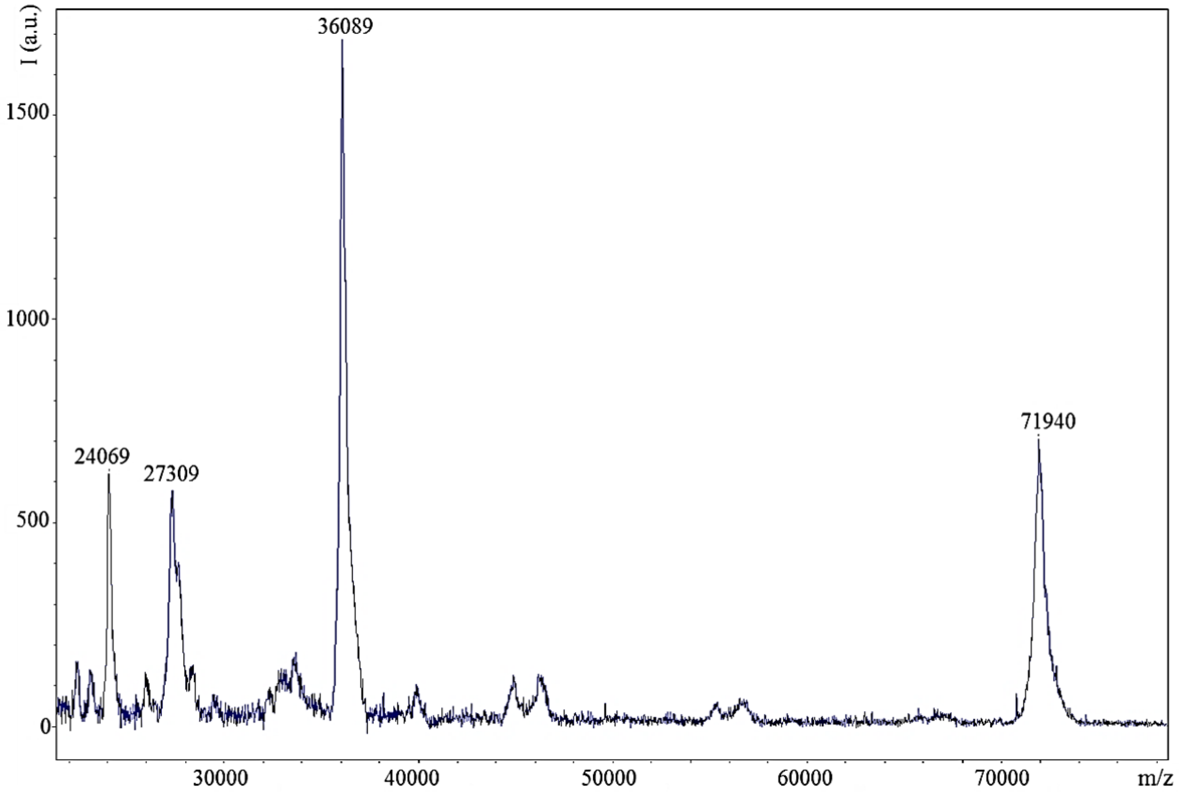

Figure S3: MALDI-TOF of the H3AH3 ELR modified with azide group.

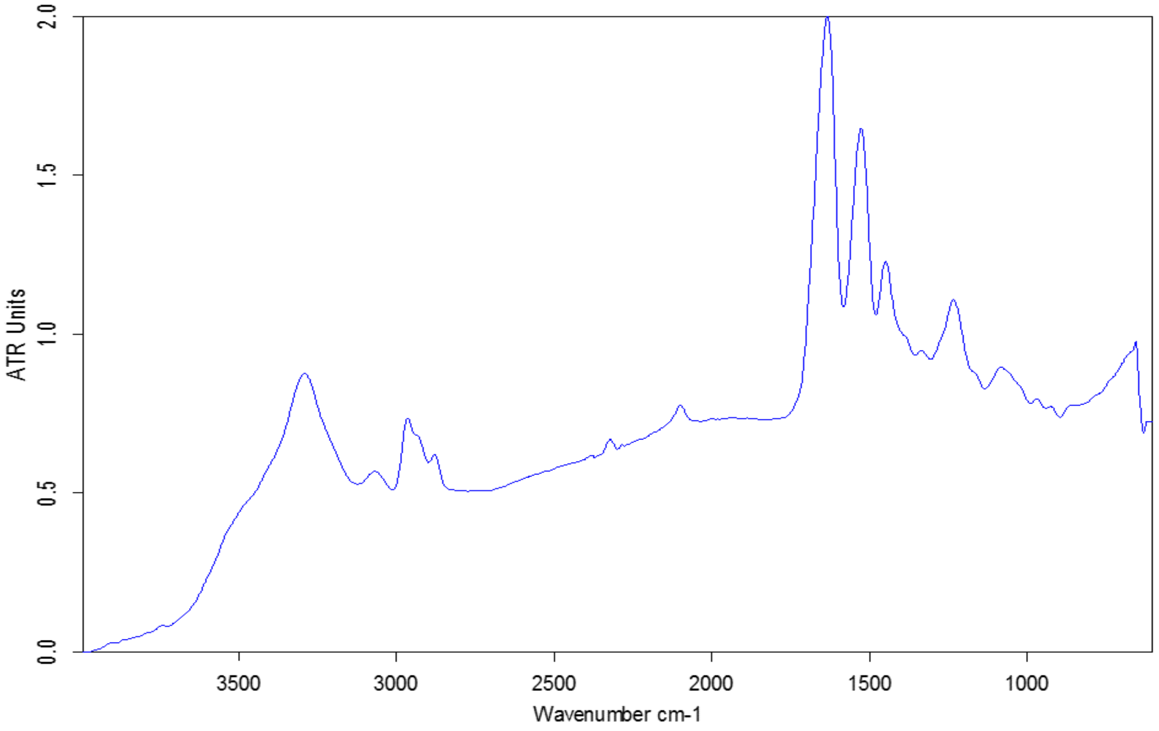

Figure S4: ATR of the H3AH3 ELR modified with azide group.

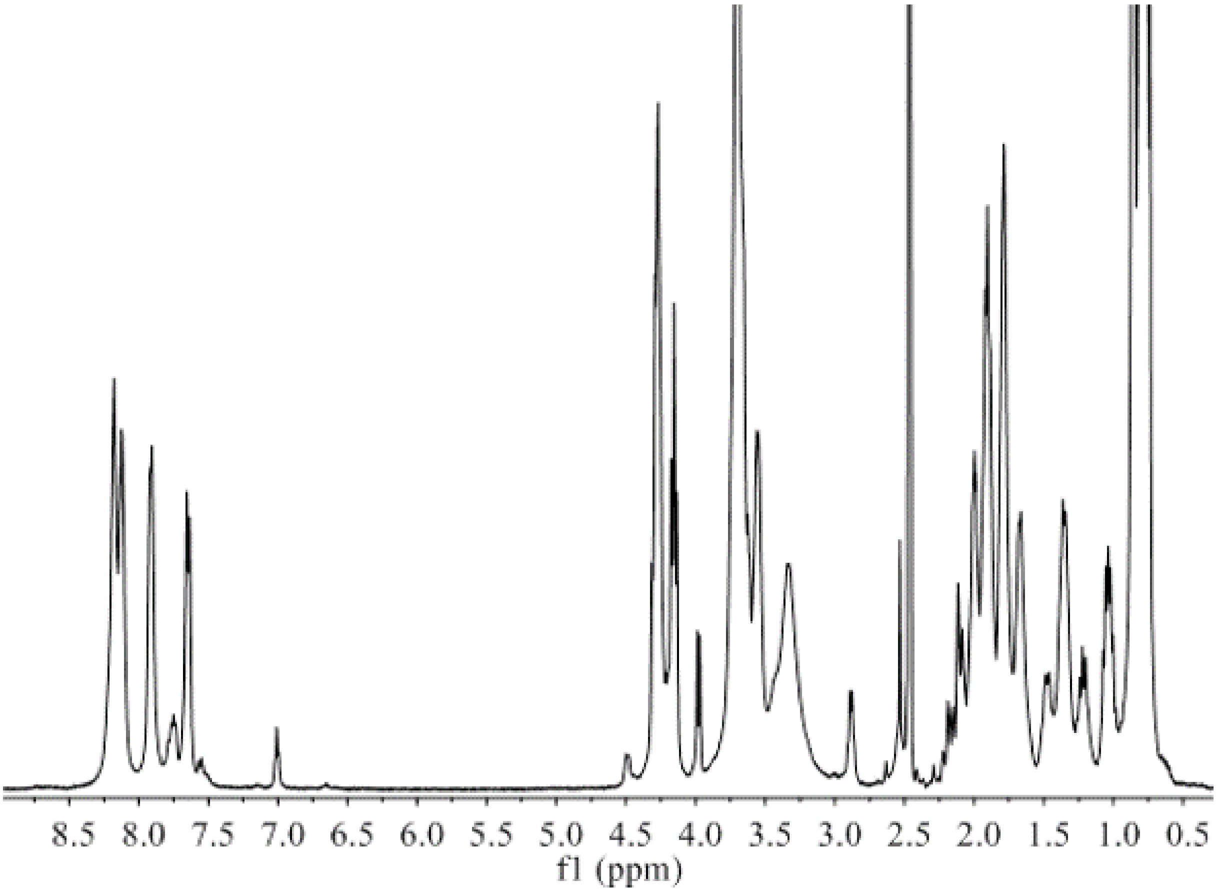

Figure S5: ^{1}H NMR in DMSO-d_6 of the IK24 ELR modified with cyclooctyne group.

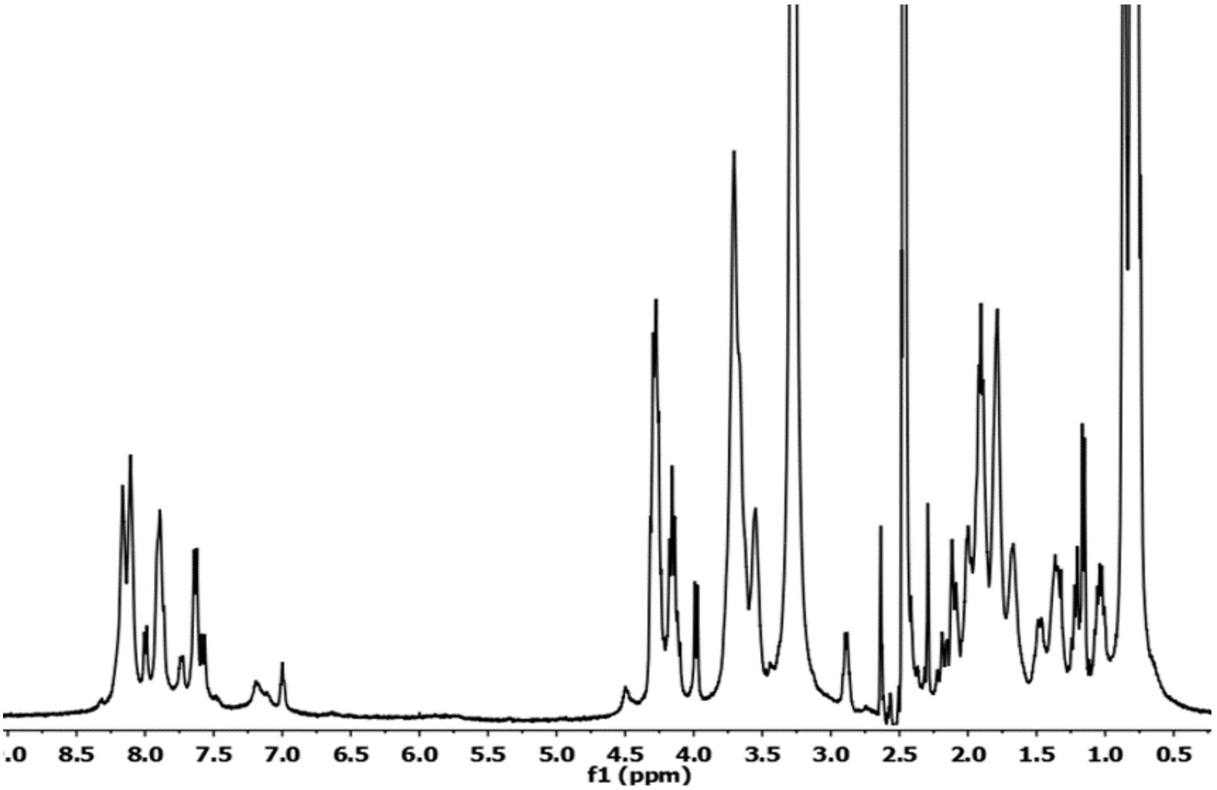

Figure S6: ^{1}H NMR in DMSO-d_6 of the H3AH3 ELR modified with cyclooctyne group.

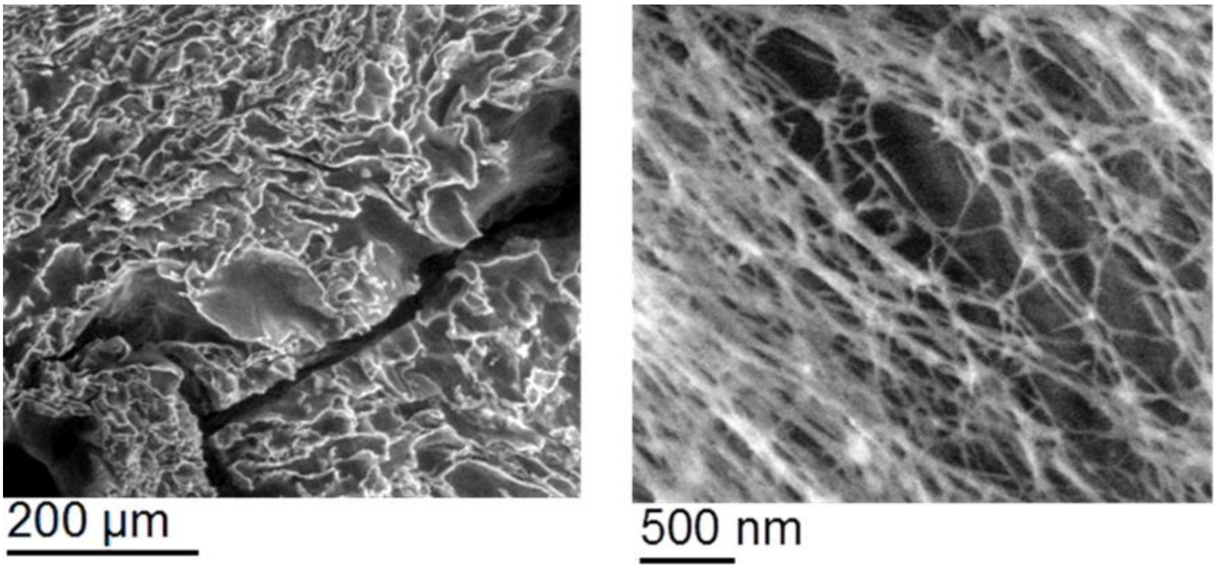

Figure S7: IK24 ELR hydrogel morphology observed by SEM.

Table S1: Elemental composition of the hydrogels after mineralization from EDXS. n.d. = not detected (detection limit of the instrument is 0.5%).

**Element**	**IK24**			**H3AH3**		
	4 days	8 days	14 days	4 days	8 days	14 days
**C *K***	33.59	20.72	26.77	27.08	39.17	32.04
**N *K***	06.70	n.d.	n.d.	07.00	n.d.	01.90
**O *K***	35.16	50.13	35.51	47.42	37.52	46.54
**P *K***	09.30	11.63	12.82	08.15	8.70	06.40
**K *K***	04.40	04.37	n.d.	01.21	3.95	n.d.
**Cl *K***	n.d.	n.d.	5.41	n.d.	n.d.	01.72
**Ca *K***	10.84	13.15	19.48	09.12	10.65	11.40

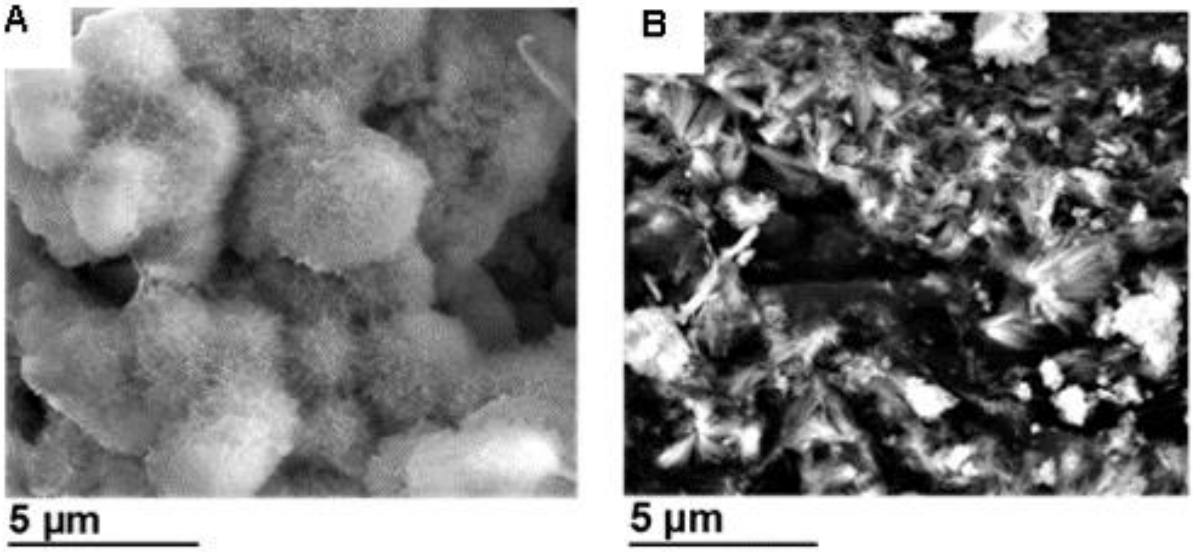

Figure S8: SEM of (A) Spherical- and (B) plate- like HA formed by the H3AH3 hydrogel after incubation for 14 days.
